# Correlation of *MLH1* and *MGMT* methylation levels between peripheral blood leukocytes and colorectal tissue DNA samples in colorectal cancer patients

**DOI:** 10.3892/ol.2013.1543

**Published:** 2013-08-23

**Authors:** XIA LI, YIBAINA WANG, ZUOMING ZHANG, XIAOPING YAO, JIE GE, YASHUANG ZHAO

**Affiliations:** Department of Epidemiology, Public Health College, Harbin Medical University, Harbin, Heilongjiang 150081, P.R. China

**Keywords:** colorectal cancers, *MLH1*, *MGMT*, methylation

## Abstract

CpG island methylation in the promoter regions of the DNA mismatch repair gene mutator L homologue 1 (*MLH1*) and DNA repair gene O^6^-methylguanine-DNA methyltransferase (*MGMT*) genes has been shown to occur in the leukocytes of peripheral blood and colorectal tissue. However, it is unclear whether the methylation levels in the blood leukocytes and colorectal tissue are correlated. The present study analyzed and compared the levels of *MGMT* and *MLH1* gene methylation in the leukocytes of peripheral blood and colorectal tissues obtained from patients with colorectal cancer (CRC). The methylation levels of *MGMT* and *MLH1* were examined using methylation-sensitive high-resolution melting (MS-HRM) analysis. A total of 44 patients with CRC were selected based on the *MLH1* and *MGMT* gene methylation levels in the leukocytes of the peripheral blood. Corresponding colorectal tumor and normal tissues were obtained from each patient and the DNA methylation levels were determined. The correlation coefficients were evaluated using Spearman’s rank test. Agreement was determined by generalized κ-statistics. Spearman’s rank correlation coefficients (r) for the methylation levels of the *MGMT* and *MLH1* genes in the leukocytes of the peripheral blood and normal colorectal tissue were 0.475 and 0.362, respectively (P=0.001 and 0.016, respectively). The agreement of the *MGMT* and *MLH1* gene methylation levels in the leukocytes of the peripheral blood and normal colorectal tissue were graded as fair and poor (κ=0.299 and 0.126, respectively). The methylation levels of *MGMT* and *MLH1* were moderately and weakly correlated between the patient-matched leukocytes and the normal colorectal tissue, respectively. Blood-derived DNA methylation measurements may not always represent the levels of normal colorectal tissue methylation.

## Introduction

DNA methylation is a significant regulator of gene transcription, and its role in carcinogenesis has become a topic of considerable interest in the last few years. DNA cytosine methylation has been widely studied, with investigations often focusing on the methylation level of CpG dinucleotides in promoter regions that usually have higher concentrations of CpGs, known as CpG islands (1). The methylation of normally unmethylated CpG islands in the promoter regions of DNA repair genes is correlated with a loss of expression of these genes (2–5), which occurs in the early stages of colorectal cancer (CRC) development (6–8).

Methylation of DNA mismatch repair gene mutator L homologue 1 (*MLH1*) and DNA repair gene O^6^-methylguanine-DNA methyltransferase (*MGMT*), is known to cause high-degree microsatellite instability (MSI-H) (4) and guanine to adenine mutations in *KRAS*, *TP53*(9) and *PIK3CA*(6), respectively. Methylation of the *MLH1* promoter has been reported in sporadic MSI tumors and is associated with the loss of protein expression (4,7,8). *MGMT* encodes a DNA repair enzyme that removes the mutagenic adduct from O^6^-methylguanine (10). Alterations in the *MGMT* gene impair the ability of the *MGMT* protein to remove the mutagenic adduct from O^6^-methylguanine, thereby increasing the mutation rate (6,9) and the risk of cancer (11).

To date, studies with regard to the methylation of genes have mainly focused on the methylation level of tumor tissues (12–15). Although the majority of CpG islands are unmethylated in normal tissues, the methylation changes of a small subset of genes may be observed under physiological conditions in normal colonic mucosa (16–18). In a previous study, samples of colorectal mucosa collected from healthy individuals undergoing screening colonoscopies were analyzed for *MLH1* and *MGMT* promoter methylation and low background methylation levels were subsequently identified (0.1–18.8%) (19). The results of another study that analyzed 13 types of normal somatic tissues, placenta, sperm and an immortalized cell line, indicated that ~18% of the genomic regions exhibited a significant difference in DNA methylation levels among the 16 tissues analyzed and were classified as tissue-specific differentially methylated regions (20). Furthermore, studies have focused on the detection of methylated DNA in peripheral blood and normal tissues (21,22), and *MLH1*(23–26) and *MGMT*(27–29) gene methylation has been reported in peripheral blood leukocytes.

Previous studies (16–18) on DNA methylation typically used paired tumor and normal surrounding tissues from cancer-bearing individuals. To the best of our knowledge, no studies with regard to the correlation between DNA methylation levels in peripheral blood leukocytes and colorectal tissue specimens, including colorectal tumor and normal colorectal tissues, from each matched patient have been published. Therefore, the present study aimed to determine whether there was a correlation between the *MLH1* and *MGMT* methylation levels in patient-matched peripheral blood leukocytes and colorectal tissue DNA samples.

## Materials and methods

### Individuals and study samples

Samples (5 ml) of peripheral blood and colorectal tumor and normal tissues were obtained from 44 patients with CRC who underwent surgery in the Department of Surgery of the Tumor Hospital (Harbin, China). Informed consent was obtained from the surgeons and patients. No patients were administered pre-operative radiation or chemotherapy. The normal colorectal mucosa specimens were obtained from colorectal tissues at the margins of the resected specimens (≥10 cm away from the tumor). Approval for this study was obtained from the Human Subjects Committee, Harbin Medical University.

The methylation status of *MLH1* and *MGMT* was examined in the peripheral blood leukocyte DNA of the CRC cases. Based on the *MLH1* and *MGMT* methylation results that were detected in the peripheral blood leukocytes (0% methylation as a cut-off value), 19 individuals with methylation of either gene were selected as positive subjects and another 25 individuals without methylation for both genes were selected as negative subjects.

### Sodium bisulfite conversion

The genomic DNA was extracted from the blood samples and colorectal tissue specimens, including colorectal tumor and normal colorectal tissues, using a TIAN-amp Genomic DNA kit (Tiangen, Beijing, China), according to the manufacturer’s instructions. Sodium bisulfite conversion of the genomic DNA was performed as described previously (29). DNA (1 μg) was bisulfite-modified using the EZ DNA Methylation-Gold kit (Zymo Research, Orange County, CA, USA). The eluted DNA (10 μl volume) was used for the methylation-sensitive high-resolution melting (MS-HRM) analysis.

### Methylation analysis

Methylation of the *MGMT* and *MLH1* promoter was assessed using MS-HRM (30). The primers used were those designed by Wojdacz and Dobrovic (30). For *MGMT*, the published primer sequences (31) and the designed sequences for *MLH1* were 5′-TTTTTTTAGGAGTGAAGGAGG-3′ and 5′-AACRCCACTACRAAACTAAA-3′. The reactions were performed in 96-well LightCycler^®^ 480 plates (Roche, Mannheim, Germany) using the LightCycler 480 High Resolution Melting Master mix, which contains a DNA intercalating dye in a final volume of 10 μl. The reaction mixture contained 1× LightCycler 480 High Resolution Melting Master mix, 200 nmol/l each primer and 1 μl bisulfite-modified DNA, with 3.0 mmol/l final MgCl_2_ for *MLH1* and *MGMT*. Each reaction was performed in duplicate. The cycling conditions that were used for the two assays were as follows: SYBR Green 1 detection format; 1 cycle at 95°C for 10 min, 50 cycles at 95°C for 10 sec, a touch down from 64°C to 58°C for 10 sec (1°C/cycle) and 72°C for 20 sec, followed by an HRM step at 95°C for 1 min, 40°C for 1 min, 74°C for 5 sec and continuous acquisition to 90°C at 25 acquisitions per 1°C. Each plate included multiple water blanks for a negative control. Methylated and unmethylated genomic templates were used to calibrate the quantitative measurements of methylation. CpGenome Universal Methylated DNA (Zymo Research) was used as 100% methylated control DNA. CpGenome Universal Unmethylated DNA (Zymo Research) was used as unmethylated control DNA. Methylation standards were constructed by diluting 100% methylated bisulfite-modified control DNA in a pool of bisulfite-modified unmethylated control DNA at levels of 50, 25, 5 and 1%. These standards were included in each experimental run. Based on the standard curves, the patient data were classified into various methylation categories by two independent observers. Disagreements were settled by consensus or a third review for adjudication.

### Statistical analysis

The data were analyzed using non-parametric Friedman and χ^2^ tests for the comparison of methylation levels in the peripheral blood leukocyte, colorectal tumor and normal colorectal tissue DNA. Spearman’s rank correlation coefficient was used for analyzing the associations of the methylation levels between the three groups. The magnitude of the correlation was specified as weak (0.00–0.39) moderate (0.40–0.79) and strong (0.80–1.00). Agreements of the levels of methylation between the peripheral blood leukocytes and normal colorectal tissues were determined using generalized weighted κ-statistics (32) Agreement was classified as excellent (κ>0.80), good (0.61≤κ≤0.80), moderate (0.41≤κ≤0.60), fair (0.21≤κ≤0.40) or poor (κ<0.20). SPSS (version 16.0; SPSS, Inc., Chicago, IL, USA) was used to analyze the data. P≤0.05 was considered to indicate a statistically significant difference.

## Results

### Patient characteristics

A total of 44 patients, 29 males and 15 females (mean age, 55 years; range, 28–79 years), were selected for the present study. The basic characteristics of the patients are shown in Table I.

### Comparing methylation status in patient-matched peripheral blood leukocyte and colorectal tissue DNA

**Figure 1:**
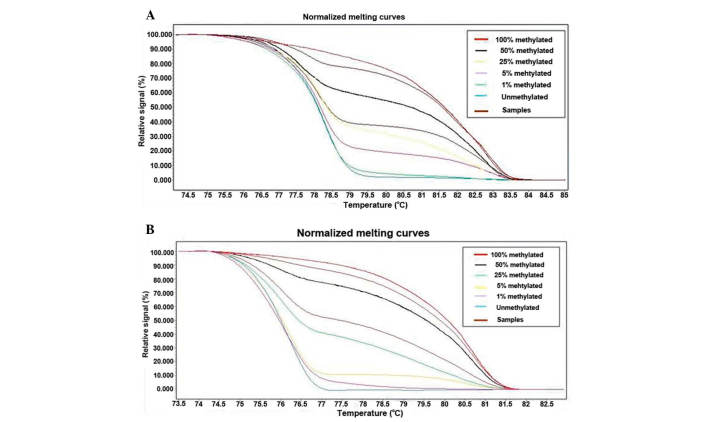
Normalized methylation-sensitive high-resolution melting (MS-HRM) standard curves of *MLH1* and *MGMT* in CRC. (A) Profile of fluorescence obtained at the melting temperature for serial dilutions of methylated DNA (100-0%) and the melting plot for the *MGMT* gene. (B) Profile of fluorescence obtained at the melting temperature for serial dilutions of methylated DNA (100-0%) and the melting plot for the *MLH1* gene. *MLH1*, DNA mismatch repair gene mutator L homoloue 1; *MGMT*, DNA repair gene O^6^-methylguanine-DNA methyltransferase; CRC, colorectal cancer.

The methylation analysis results of the 44 patients are shown in Table II and illustrated in Fig. 1A and 1B. Differences in the levels of *MGMT* and *MLH1* methylation were examined in patient-matched peripheral blood leukocyte, colorectal tumor and normal colorectal tissue DNA (Table II). There were no significant differences in the levels of *MGMT* and *MLH1* methylation between the three groups (Friedman test, P=0.260 and P=0.464, respectively).

Various cut-off methylation levels were used for the analysis (Table III). The level of methylation was classified as positive at a cut-off value of 0–1% methylation and no statistical significant differences were observed in the levels of *MGMT* and *MLH1* methylation among the three groups (Table III). When a level of methylation of >5% was classified as positive, there was a significant difference in the levels of *MGMT* methylation among the three groups (P=0.014; χ^2^ test), but no significant difference in the levels of *MLH1* methylation (P=0.251; χ^2^ test). Further analysis revealed that a significant difference in *MGMT* methylation existed between colorectal tumor tissue DNA and leukocytes or normal colorectal tissue DNA (P=0.013 and P=0.035, respectively; χ^2^ test), but not between leukocyte and normal colorectal tissue DNA (P=0.645; χ^2^ test).

### Spearman rank correlation coefficients

Positive correlations were observed between the peripheral blood leukocyte and normal colorectal tissue DNA in the levels of *MGMT* and *MLH1* methylation (r=0.475, P=0.001 and r=0.362, P=0.016, respectively). However, there were no positive correlations between colorectal tumor tissue and peripheral blood leukocyte or normal colorectal tissue DNA, based on the methylation levels of the assessments of the two genes (Table IV).

### Agreement

The agreement between the peripheral blood leukocyte and normal colorectal tissue DNA with CRC on the levels of *MGMT* and *MLH1* methylation were calculated using κ coefficients (Table V). The agreement of the MGMT gene methylation levels in the leukocytes of the peripheral blood and normal colorectal tissue was graded as fair (κ=0.299). The agreement of the MLH1 gene methylation levels in the leukocytes of the peripheral blood and normal colorectal tissue was graded as poor (κ=0.126) (Table V).

## Discussion

DNA promoter methylation has previously been shown to be a well-characterized event in tumor biology and has been extensively documented in CRC (33,34). However, few studies have compared DNA promoter methylation in DNA from various patient-matched sources. The present results revealed that the levels of *MLH1* and *MGMT* methylation were not significantly different in patient-matched peripheral blood leukocyte, colorectal tumor tissue and normal colorectal tissue DNA as original semi-quantitatively rank data. Since low background methylation levels have previously been reported for various genes in normal samples (35,36), several studies have used 0.1–10% as a cut-off for the scoring criteria of gene methylation using the MS-HRM assay (31,37–40) and there has not been a unified standard to define methylation. Therefore, in the present study, in order to identify the various methylation levels between cancer and normal samples, a range of cut-off methylation levels were used. When samples with >5% methylation were considered as methylated, distinctive *MGMT* gene promoter methylation levels were identified between colorectal tumor tissue and leukocyte or normal colorectal tissue DNA, but no significant differences were observed between leukocyte and normal colorectal tissue DNA. Thus, in samples containing >5% methylation, the analysis of the *MGMT* gene appeared to increase the sensitivity for discriminating cancer from normal colorectal tissues or leukocytes.

Previous studies have focused on DNA methylation measured in the leukocytes (41,42) or the normal mucosa tissues (17,43,44), rarely reporting the correlation between the two. Ally *et al* reported significant positive correlations between the estrogen receptor-α methylation index in leukocytes and normal colonic tissue in CRC patients (r=0.570; P=0.003) (45). However, the samples were not case matched. Spearman rank correlations were performed to investigate the correlation of the *MLH1* and *MGMT* methylation levels in patient-matched peripheral blood leukocyte, colorectal tumor and normal colorectal tissue DNA samples. The most significant positive correlations were observed between the leukocyte and normal colorectal tissue DNA for methylation detection of *MGMT* and *MLH1* (r=0.475 and r=0.362, respectively).

In human studies, ethical and practical barriers may make it difficult or impossible to collect specimens from the target tissue. Hence, the use of surrogate samples, including DNA derived from easily accessible peripheral blood, is widely accepted when the target tissue is unobtainable. Several studies with regard to DNA methylation biomarkers tested in leukocytes suggest the suitability of epigenetic biomarkers for the detection of several cancers relative to controls (46–48). Widschwendter *et al* identified that particular methylation patterns in peripheral blood DNA may serve as surrogate markers for the risk of breast cancer (49). To investigate the use of blood as a surrogate for DNA methylation in tissues, the present study measured the *MGMT* and *MLH1* gene methylation levels in the leukocytes of the peripheral blood and normal colorectal tissue, which were graded as fair and poor (κ=0.299 and 0.126, respectively); therefore, blood-derived DNA methylation level measurements may not always represent the levels of target colorectal tissue methylation (50). While all somatic cells in a given individual are genetically identical, differing cell types form highly distinct anatomical structures and perform a wide range of disparate physiological functions (51). It has been conjectured that during tissue differentiation and development, transcription-relevant control regions in the genome become selectively de- or upmethylated to enable the transcription of a restricted set of genes within a given tissue (52). It is also plausible that methylation patterns in DNA obtained from blood may be more ‘plastic’ compared with that of other tissues, due to the close proximity of the blood to environmental influences, such as nutrition and smoking (50).

A limitation of the present study is the fact that the subjects that were selected. The methylation results may not reflect the natural frequencies of methylation in leukocytes or colorectal tissues. Another limitation of the study is a lack of inclusion of healthy controls. Studies using cancer subjects may not exclude the possibility of disseminated tumor cells or the effect the disease itself may have on the systemic methylation status in leukocytes and normal colorectal tissues. Further studies with disease-free individuals and an investigation of tumor suppressor gene methylation are required to clarify this issue.

In summary, the correlation of *MGMT* and *MLH1* methylation levels between patient-matched leukocytes and normal colorectal tissues was classified as moderate and weak, respectively. Blood-derived DNA methylation measurements may not always represent the levels of normal colorectal tissue methylation.

## Figures and Tables

**Table I:** Demographic and clinical characteristics of 44 CRC patients.

Patient characteristics	No. of cases (%)
Age, years	
≤60	26 (59.1)
>60	18 (40.9)
Total	44 (100.0)
Gender	
Female	15 (34.1)
Male	29 (65.9)
Tumor location	
Proximal colon^a^	8 (18.2)
Distal colon^b^	6 (13.6)
Rectum	30 (68.2)
Tumor stage	
I	3 (6.8)
II	22 (50.0)
III	18 (40.9)
IV	1 (2.3)

a Proximal colon includes the cecum through the transverse colon.

b Distal colon includes the descending colon through the rectum.

CRC, colorectal cancer.

**Table II:** MS-HRM assay of peripheral blood and colorectal tissue samples of CRC patients.

	*MGMT*, n			*MLH1*, n		
		
Frequencies of methylation (%)	Leukocytes	Normal tissue	Tumor tissue	Leukocytes	Normal tissue	Tumor tissue
0	37	35	31	32	30	31
0–1	0	2	1	0	2	2
1–5	5	4	2	7	1	2
5–25	0	3	3	2	9	5
25–50	1	0	0	3	1	2
50–100	1	0	2	0	0	1
100	0	0	5	0	1	1

MS-HRM, methylation-sensitive high-resolution melting; CRC, colorectal cancer; *MGMT*, DNA repair gene O^6^-methylguanine-DNA methyltranserferase; *MLH1*, DNA mismatch repair gene mutator L homologue 1.

**Table III:** MS-HRM assay of peripheral blood and colorectal tissue samples of CRC patients using various cut-off values.

	*MGMT*					*MLH1*			
					
Cut-off (%)	Leukocytes, n (%)	Normal tissue, n (%)	Tumor tissue, n (%)	P-value*	P-value*	Leukocytes, n (%)	Normal tissue, n (%)	Tumor tissue, n (%)	P-value*
0	7 (15.9)	9 (20.5)	13 (29.5)	0.282	-	12 (27.3)	14 (31.8)	13 (29.5)	0.897
1	7 (15.9)	7 (15.9)	12 (27.3)	0.302	-	12 (29.5)	12 (29.5)	11 (25.0)	0.962
5	2 (4.5)	3 (6.8)	10 (22.7)	0.014	0.645^a^, 0.035^b^, 0.013^c^	5 (11.4)	11 (25.0)	9 (20.5)	0.251

* χ^2^ test; leukocytes vs. normal tissue;

b tumor tissue vs. normal tissue; and

c tumor tissue vs. leukocytes.

MS-HRM, methylation-sensitive high-resolution melting; CRC, colorectal cancer; *MGMT*, DNA repair gene O^6^-methylguanine-DNA methyltranserferase; *MLH1*, DNA mismatch repair gene mutator L homologue 1.

**Table IV:** Spearman’s rank correlation coefficients (P-values) of *MGMT* and *MLH1* methylation levels in case-matched DNA with CRC.

DNA source	Leukocytes	Normal tissues	Tumor tissues
*MGMT*			
Leukocytes	-	0.475 (0.001)	−0.033 (0.833)
Normal tissues	0.475 (0.001)	-	0.025 (0.873)
Tumor tissues	−0.033 (0.833)	0.025 (0.873)	-
*MLH1*			
Leukocytes	-	0.362 (0.016)	0.215 (0.161)
Normal tissues	0.362 (0.016)	-	0.293 (0.054)
Tumor tissues	0.215 (0.161)	0.293 (0.054)	-

*MGMT*, DNA repair gene O^6^-methylguanine-DNA methyltranserferase; *MLH1*, DNA mismatch repair gene mutator L homologue 1; CRC, colorectal cancer.

**Table V:** Frequencies of *MGMT* and *MLH1* methylation in the peripheral blood and the normal colorectal tissues.

	Frequencies of normal colorectal tissues methylation (%)							
		
Frequencies of blood methylation (%)	0	0–1	1–5	5–25	25–50	50–100	100	n
*MGMT*								
0	32	2	3	0	0	0	0	37
0–1	0	0	0	0	0	0	0	0
1–5	3	0	1	1	0	0	0	5
5–25	0	0	0	0	0	0	0	0
25–50	0	0	0	1	0	0	0	1
50–100	0	0	0	1	0	0	0	1
100	0	0	0	0	0	0	0	0
n	35	2	4	3	0	0	0	44
κ	0.299 (P=0.002)							
*MLH1*								
0	24	2	1	5	0	0	0	32
0–1	0	0	0	0	0	0	0	0
1–5	5	0	0	2	0	0	0	7
5–25	1	0	0	1	0	0	0	2
25–50	0	0	0	1	1	0	1	3
50–100	0	0	0	0	0	0	0	0
100	0	0	0	0	0	0	0	0
n	30	2	1	9	1	0	1	44
κ	0.126 (P=0.098)							

κ: The κ statistics are parameters of agreement that take chance agreement into account.

*MGMT*, DNA repair gene O^6^-methylguanine-DNA methyltranserferase; *MLH1*, DNA mismatch repair gene mutator L homologue 1.
